# Where the final day is spent: patterns in place of death among U.S. adults with alcoholic liver cirrhosis, 1999–2023

**DOI:** 10.1097/MS9.0000000000005447

**Published:** 2026-07-30

**Authors:** Asad Ali Ahmed Cheema, Syed Sarmad Javaid, Fabeha Zafar, FNU Samina, Muhammad Sufian, Muhammad Hamza Saghir, Muhammad Zohaib Qasim, Zainab Pervaiz, Warda Nasir, Muhammad Hashir Khan, Ali Ghaffar, Hamza Bashir, Muhammad Shikaib Shabbir

**Affiliations:** a Department of Medicine, International School of Medicine, International University of Kyrgyzstan, Bishkek, Kyrgyzstan; b Department of Medicine, University of Mississippi Medical Center, Jackson, USA; c Department of Medicine, Dow University of Health Sciences, Karachi, Pakistan; d Department of Medicine, Jinnah Sindh Medical University, Karachi, Pakistan; e Department of Medicine, Aga Khan University, Karachi, Pakistan; f Department of Medicine, Akhtar Saeed Medical and Dental College, Lahore, Pakistan; g Department of Medicine, Quaid-e-Azam Medical College, Bahawalpur, Pakistan; h Department of Medicine, CMH Lahore Medical College, Lahore, Pakistan; i Department of Medicine, Faisalabad Medical University, Faisalabad, Pakistan; j Department of Medicine, Nishtar Medical College & Hospital, Multan, Pakistan; k Department of Medicine, Ameer-ud-Din Medical College, Lahore, Pakistan; l Department of Medicine, University of California Riverside SOM, Riverside, California, USA

**Keywords:** alcoholic liver cirrhosis, CDC WONDER, mortality trends, palliative care, place of death

## Abstract

**Background:**

Alcoholic liver cirrhosis (ALC) remains a leading cause of liver-related mortality in the United States (U.S.); however, national data on place of death and sociodemographic patterns are limited. This study aimed to evaluate 25-year national trends in ALC-related mortality and place of death and to examine demographic and geographic predictors shaping end-of-life outcomes.

**Methods:**

Using CDC WONDER death certificate data and the National Center for Health Statistics individual-level mortality files, we analyzed deaths among adults aged ≥25 years (1999–2023). Place of death was categorized as hospital, home, hospice, or nursing home/long-term care; emergency/outpatient deaths were examined separately.

**Results:**

From 1999 to 2023, 302 815 ALC-related deaths occurred. Inpatient deaths fell from 65.2 to 48.6%, while home deaths rose from 20.7 to 30.2%. Hospice/nursing facility deaths increased to 23.7% in 2015, then declined to 18.3% in 2023; emergency/outpatient deaths decreased from 4.1 to 2.8%. Ages 50–64 accounted for most deaths, and younger adults had the highest inpatient proportion. In multivariable models, sex, race/ethnicity, and urbanization were significant: men, racial/ethnic minorities, and metropolitan residents were more likely to die in inpatient or emergency settings.

**Conclusion:**

ALC-related deaths shifted from inpatient facilities toward home and the combined hospice/long-term care facility category, while sociodemographic differences in place of death suggest uneven end-of-life care pathways and support efforts to improve equitable palliative and supportive care planning.

## Introduction

Alcoholic liver cirrhosis (ALC) represents the end stage of alcohol-associated liver disease (ALD) and is a leading cause of liver-related mortality in the United States (U.S.). As of 2017, over 2 million Americans were living with alcohol-associated cirrhosis, and ALC-related deaths tripled between 1999 and 2019^[^1,2^]^. The burden intensified during the COVID-19 pandemic, with alcohol use and associated mortality spiking sharply^[^3^]^. Despite this burden, limited attention has been paid to end-of-life trajectories in this population. Given the rising mortality burden, understanding how and where patients with ALC spend their final days is essential for evaluating end-of-life care needs and identifying gaps in palliative support.

HIGHLIGHTS

- National trends in alcoholic liver cirrhosis-related mortality and in place of death were analyzed over 25 years.
- Inpatient deaths declined, while home and hospice deaths increased over time.
- Hospice deaths peaked in 2015 and have declined in recent years.
- Younger adults, men, and minorities were less likely to die at home or in hospice.
- Findings highlight disparities and underscore the need for equitable access to palliative care.

Place of death has emerged as a key indicator of the quality, accessibility, and equity of end-of-life care. Dying at home or in hospice is associated with greater comfort, caregiver satisfaction, and alignment with patient preferences across many chronic illnesses, including cancer and heart failure^[^4,5^]^. Individuals with cirrhosis, particularly those with alcohol-related liver disease, often face a range of clinical and structural barriers that hinder timely hospice referral and advance care planning^[^6^]^. Stigma, late-stage presentation, and unpredictable disease progression often result in deaths occurring in emergency or acute care settings rather than supportive environments. However, no national study to date has examined where patients with ALC die or how this varies by demographics.

In the U.S., palliative care services have expanded substantially but remain unevenly distributed. The 2024 Center to Advance Palliative Care Serious Illness Scorecard reports that 83.6% of hospitals with 50 or more beds and 96.2% of hospitals with 300 or more beds now have established palliative care services. Yet access remains limited in rural and privately owned hospitals, and community-based services remain underdeveloped, particularly among racial and ethnic minorities. In parallel, the epidemiology of liver transplantation has shifted markedly toward alcohol- and metabolism-related etiologies^[^7^]^. Younossi *et al* in 2024 found that ALD and metabolic dysfunction–associated steatotic liver disease (MASLD) have become the leading causes of liver transplantation in the U.S. The proportion of transplants attributed to ALD nearly doubled from 23% in 2013 to 48% in 2022, while those due to MASLD rose from 19 to 27% over the same period. Conversely, hepatitis C-related cases declined sharply from 28 to 4%^[^8^]^. A bicontinental registry study by Younossi *et al* in 2025 confirmed ALD and MASLD as the fastest-growing global indications, and Feng *et al* similarly reported rising MASLD prevalence by 11.2% with declining viral hepatitis^[^9,10^]^. Together, these trends highlight the widening inequities in access to palliative and advanced liver care.

This study aims to fill that gap by analyzing national U.S. trends in ALC-related mortality and place of death over 25 years, using data from the Centers for Disease Control and Prevention’s Wide-ranging Online Data for Epidemiologic Research (CDC WONDER) database. By stratifying results by age, sex, race/ethnicity, and geographic region, we aim to identify patterns and disparities in both mortality burden and end-of-life care access, as well as temporal shifts across the study period. These insights may inform policy, support patient-centered care planning, and guide efforts to improve equitable delivery of palliative services for individuals affected by alcohol-associated cirrhosis.

## Methods

### Data source and extraction

We utilized publicly available mortality data from the National Center for Health Statistics, accessed through the CDC WONDER database, covering 1999–2023. Our analysis focused on decedents aged 25 years and older with ALC listed as the underlying cause of death, identified using the International Classification of Diseases, Tenth Revision (ICD-10) code K70.3. The underlying cause was determined by the certifying physician as the principal condition initiating the sequence of events leading to death. As the dataset was de-identified and publicly accessible, this study was exempt from institutional review board oversight and informed consent requirements under 45 CFR 46.101 per US Department of Health and Human Services regulations.

### Inclusion and exclusion criteria

We applied explicit inclusion and exclusion criteria. The inclusion criteria were decedents aged >25 years with ALC as the underlying cause of death, using ICD-10 code K70.3. The exclusion criteria were deaths due to external causes (ICD-10 codes V01-Y89, including accidents, suicides, and homicides) and cases with missing demographic or place-of-death information. The final analytic sample after exclusions is shown in Supplemental Digital Content Figure 1, available at: https://links.lww.com/MS9/B320.

### Study variables

The primary outcome was place of death, categorized into four mutually exclusive settings: inpatient medical facility, outpatient medical facility/emergency room, decedent’s home, and a combined hospice/long-term care facility category. The combined hospice/long-term care facility category included deaths occurring in hospice facilities and in nursing homes or long-term care facilities. This grouping was used to maintain consistent longitudinal classification across the full 1999–2023 study period because CDC WONDER place-of-death coding changed in 2003, with the “Hospice Facility” category available only from 2003 onward. We recognize that hospice facilities and nursing homes or long-term care facilities represent different care models. Hospice facilities primarily provide end-of-life palliative care, whereas nursing homes and long-term care facilities may reflect residential, custodial, or skilled nursing care with variable hospice involvement. Therefore, this combined category was used for place-of-death classification and should not be interpreted as direct hospice enrollment, hospice quality, or equivalent care delivery across settings.

Other variables included key demographic and geographic factors: age, sex, race, Hispanic ethnicity, and urbanization. Age was grouped into 25–34, 35–49, 50–64, and 65+ years. Race was classified as White, Black, American Indian/Alaska Native, and Asian/Pacific Islander. Hispanic origin was designated as Hispanic or non-Hispanic. Urbanization was categorized using the 2013 National Center for Health Statistics (NCHS) Urban–Rural Classification Scheme for Counties and was collapsed into large metropolitan, medium/small metropolitan, and rural/nonmetropolitan categories. Large metropolitan included large central and large fringe metropolitan counties in metropolitan statistical areas of 1 million or more residents. Medium/small metropolitan included medium metropolitan counties in metropolitan statistical areas of 250 000 to 999 999 residents and small metropolitan counties in metropolitan statistical areas of less than 250 000 residents. Rural/nonmetropolitan included micropolitan and noncore counties^[^11^]^. These variables were included to assess disparities in ALC mortality based on social determinants of health.

### Statistical analysis

A single multivariable multinomial logistic regression model was used to evaluate associations between decedent characteristics and place of death. Inpatient medical facility death was used as the reference outcome. Age group, sex, race, Hispanic ethnicity, and urbanization level were entered simultaneously as covariates in the model. The prespecified reference groups were age ≥65 years, female sex, White race, non-Hispanic ethnicity, and rural residence. Records with missing place-of-death information or missing covariate data were excluded before regression analysis, and no imputation was performed. Odds ratios (ORs) and 95% confidence intervals (CIs) were reported for outpatient medical facility/emergency room, decedent’s home, and hospice/nursing facility deaths relative to inpatient medical facility deaths. Statistical significance was set at *P* < 0.05. CDC WONDER mortality data are based on national death-certificate records rather than a sample survey design. Therefore, complex survey weights were not applied to the multinomial regression. Age-standardized mortality rates were used for descriptive temporal rate analyses and Joinpoint regression, not for estimation of multinomial regression ORs.

To assess temporal mortality trends, we applied Joinpoint regression analysis (Joinpoint Regression Program v5.2.0, National Cancer Institute), using log-linear modeling to estimate annual percent change (APC) and corresponding 95% CIs. The Weighted Bayesian Information Criterion guided the selection of optimal joinpoints^[^12^]^.

We evaluated multicollinearity among predictors using variance inflation factors (VIFs), all of which were <2, indicating no significant multicollinearity. The Independence of Irrelevant Alternatives assumption was tested with the Hausman–McFadden test, which confirmed its validity in our analysis^[^13^]^. All analyses were conducted using SPSS software, version 27.0 (IBM Corp., Armonk, NY). The study followed STROBE (Strengthening the Reporting of Observational Studies in Epidemiology) guidelines^[^14^]^.

## Results

### Demographic and temporal trends in ALC-related mortality (1999–2023)

From 1999 to 2023, 302 815 deaths were attributed to ALC in the United States. Among the decedents, 217 895 (72%) were male, 270 042 (89.2%) were White, and 154 210 (51%) were adults aged 50–64 years. Most deaths occurred in the 50–64 age group across all places of death. The 35–49 group had a notable number of inpatient and outpatient deaths, while the 65+ group had a higher proportion of deaths in home and hospice/nursing settings. The 25–34 group had the lowest number of deaths in all locations. A detailed overview of where ALC patients died, stratified by key demographic features, is provided in Table 1.

**Table 1:** Aggregated data for adults for places of death by decedent characteristics for alcoholic liver cirrhosis (1999–2023).

Category	Subgroup	Total *N* (%)	Inpatient *N* (%)	Outpatient/ER *N* (%)	Home *N* (%)	Hospice/nursing *N* (%)
Hispanic origin						
	Hispanic	49 404 (16.3%)	29 180 (59.1%)	1795 (3.6%)	12 509 (25.3%)	5920 (12.0%)
	Non-Hispanic	253 411 (83.7%)	125 745 (49.6%)	6791 (2.7%)	71 882 (28.4%)	48 993 (19.3%)
Race						
	American Indian	7496 (2.5%)	5039 (67.2%)	42 (0.6%)	1562 (20.8%)	853 (11.4%)
	Asian	2608 (0.9%)	1933 (74.1%)	46 (1.8%)	532 (20.4%)	97 (3.7%)
	Black	22 669 (7.4%)	14 134 (62.3%)	820 (3.6%)	4770 (21.0%)	2945 (13.0%)
	White	270 042 (89.2%)	133 819 (49.6%)	7678 (2.8%)	77 527 (28.7%)	51 018 (18.9%)
Sex						
	Male	217 895 (72.0%)	110 443 (50.7%)	6612 (3.0%)	62406 (28.6%)	38 434 (17.6%)
	Female	84 920 (28.0%)	44 482 (52.4%)	1974 (2.3%)	21 985 (25.9%)	16 479 (19.4%)
Urbanization						
	Large metro	153 389 (50.6%)	83 084 (54.2%)	4857 (3.2%)	39 903 (26.0%)	25 545 (16.7%)
	Medium/small metro	99 863 (33.0%)	46 888 (47.0%)	2563 (2.6%)	29 534 (29.6%)	20 878 (20.9%)
	Rural	49 563 (16.4%)	24 953 (50.3%)	1166 (2.4%)	14 954 (30.2%)	8490 (17.1%)
Age groups (years)						
	25–34	5893 (1.9%)	4097 (69.5%)	158 (2.7%)	1237 (21.0%)	401 (6.8%)
	35–49	67 749 (22.4%)	40 247 (59.4%)	2821 (4.2%)	16 609 (24.5%)	8072 (11.9%)
	50–64	154 210 (51.0%)	79 562 (51.6%)	4612 (3.0%)	42 536 (27.6%)	27 500 (17.8%)
	65 +	74 963 (24.7%)	31 019 (41.4%)	995 (1.3%)	24 009 (32.0%)	18 940 (25.3%)

ER, emergency room; metro, metropolitan area; values are *N* (%) within each subgroup.

**Figure 1.**
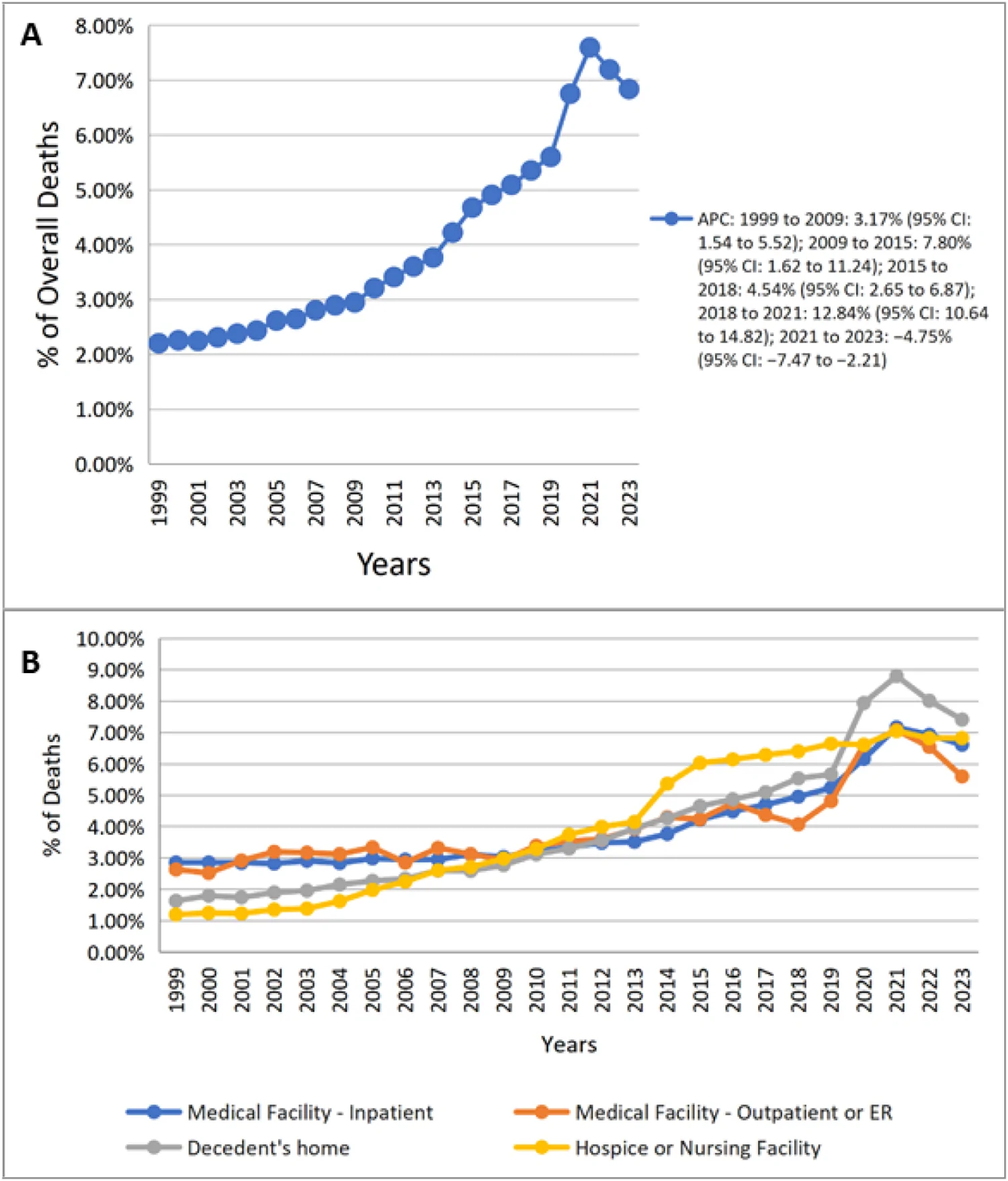
Temporal trends in alcoholic liver cirrhosis-related mortality and place of death among U.S. adults, 1999–2023. **(A)** Annual proportion of overall deaths attributed to alcoholic liver cirrhosis, with Joinpoint regression segments. Segment labels show the annual percentage change with corresponding 95% confidence intervals. (**B)** Annual distribution of place of death among adults with alcoholic liver cirrhosis-related mortality. For panel B, the denominator for each year is the total number of alcoholic liver cirrhosis-related deaths recorded in that calendar year.

ALC, alcoholic liver cirrhosis; APC, annual percentage change; CI, confidence interval; ER, emergency room.

Throughout the study period, deaths attributed to ALC increased substantially across the US. Joinpoint regression analysis identified five significant inflection points, indicating dynamic shifts in mortality trends over the 25 years. From 1999 to 2009, ALC-related mortality rose at a moderate but steady rate (APC, 3.17%; *P* < 0.05). A notable acceleration occurred from 2009 to 2015 (APC, 7.80%; *P* < 0.05). Between 2015 and 2018, the upward trend continued, albeit at a slightly reduced pace (APC, 4.54%; *P* < 0.05). The most dramatic surge was observed from 2018 to 2021, during which ALC-related deaths escalated sharply (APC, 12.84%; *P* < 0.05), signaling a critical public health concern. However, from 2021 to 2023, a reversal emerged, with mortality declining at an (APC, −4.75% *P* < 0.05; Fig. 1A).

**Figure 2.**
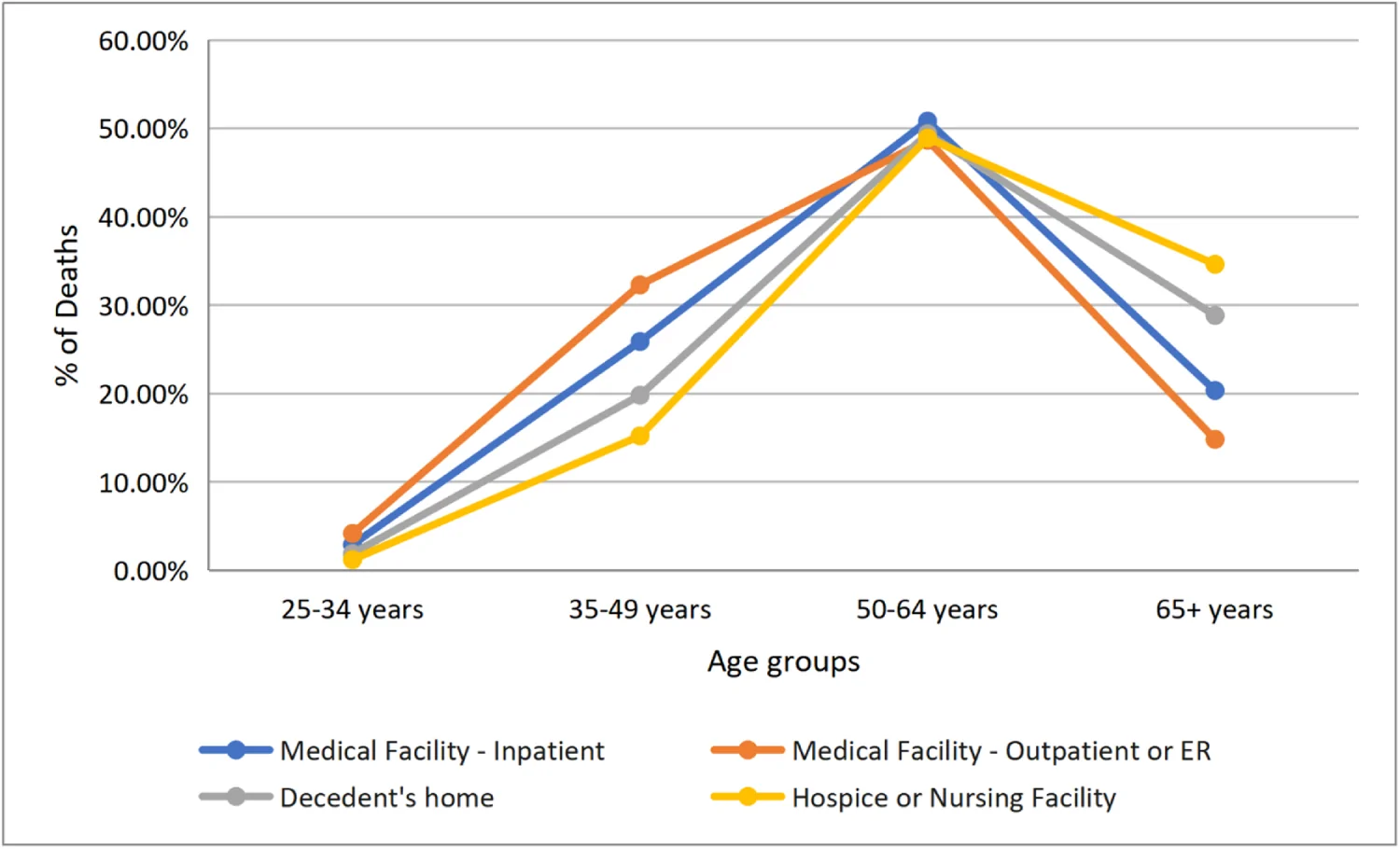
Age distribution by place of death among U.S. adults with alcoholic liver cirrhosis-related mortality, 1999–2023. Percentages are calculated within each place-of-death category for the full study period. For each line, the denominator is the total number of deaths in that place-of-death category. Values represent observed descriptive proportions.

ALC, alcoholic liver cirrhosis; ER, emergency room.

There was also a decrease in the proportion of ALC deaths in inpatient medical facilities, from 65.2% (4498 of 6895) in 1999 to 48.6% (10 407 of 21 387) in 2023, but this pattern was not linear. Figure 1B shows a significant increase in inpatient deaths during 2019–2021, coinciding with the COVID-19 pandemic, followed by a decline in 2022–2023. This indicates that the overall reduction was shaped by pandemic-related disruptions rather than a steady long-term decline. The proportion of ALC deaths in ERs decreased slightly from 4.1% (281 of 6895) in 1999 to 2.8% (597 of 21 387) in 2023. However, a significant increase in the proportion of deaths at home was recorded, rising from 20.7% (1426 of 6895) in 1999 to 30.2% (6462 of 21 387) in 2023. Over the study period, the percentage of fatalities in hospice/nursing institutions rose modestly from 10% (690 of 6895) in 1999 to 10.7% (798 of 7436) in 2003. This was followed by a substantial increase, reaching 23.7% (3466 of 14 629) in 2015. However, by 2023, the proportion declined to 18.3% (3921 of 21 387), suggesting a potential shift in end-of-life care patterns in recent years. Figure 1B displays the annual trends in locations where ALC deaths occurred. Younger adults (25–34 years) accounted for 2.6% of inpatient medical facility deaths, the lowest among all age groups. The highest proportion of hospice or nursing facility deaths was observed among those aged 50–64 years (50.1%), followed by individuals aged ≥65 years (34.5%) (Fig. 2).

### Sociodemographic predictors of place of death

**Figure 3.**
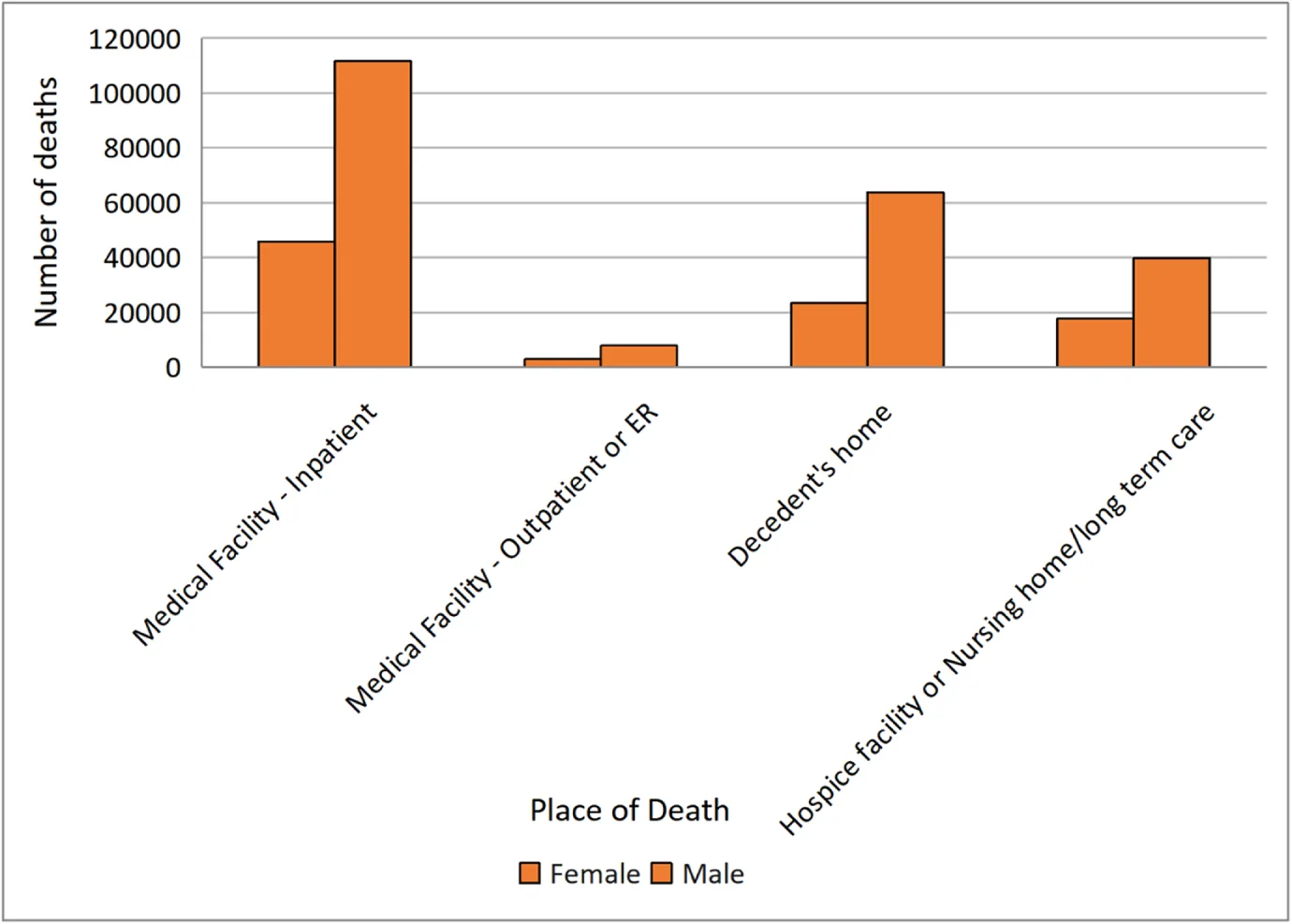
Sex-stratified number of alcoholic liver cirrhosis-related deaths by place of death among U.S. adults, 1999–2023. Bars represent observed death counts by sex and place of death. The denominator is the analytic cohort of alcoholic liver cirrhosis-related deaths with non-missing sex and place-of-death information.

ALC, alcoholic liver cirrhosis; ER, emergency room.

**Figure 4.**
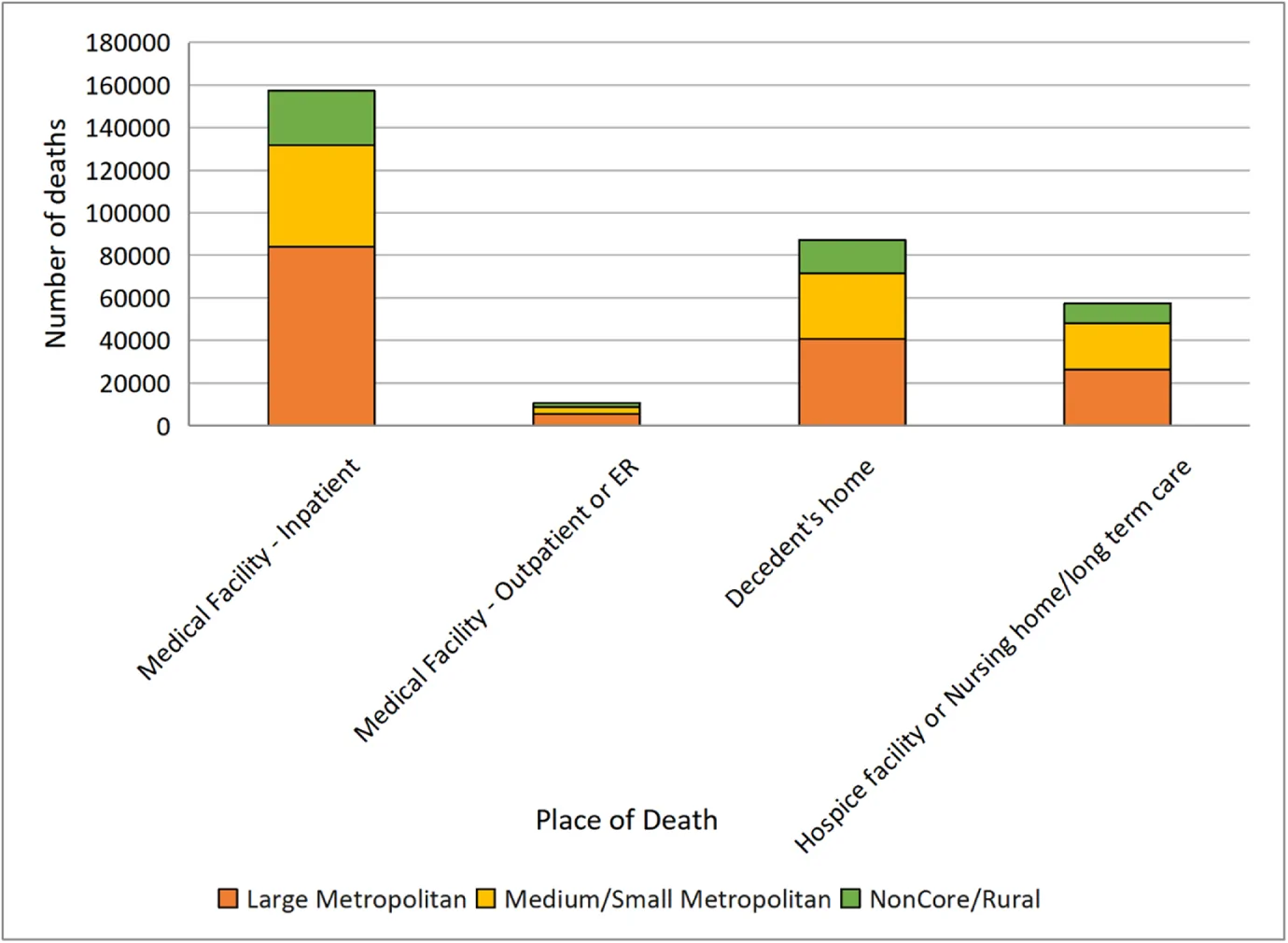
Urbanization-stratified number of alcoholic liver cirrhosis-related deaths by place of death among U.S. adults, 1999–2023. Bars represent observed death counts by urbanization level and place of death. The denominator is the analytic cohort of alcoholic liver cirrhosis-related deaths with non-missing urbanization and place-of-death information. Rural/nonmetropolitan includes micropolitan and noncore counties according to the National Center for Health Statistics Urban-Rural Classification Scheme.

ALC, alcoholic liver cirrhosis; ER, emergency room; metro, metropolitan.

Multivariable logistic regression analysis identified significant associations between decedent characteristics and the place of death, using inpatient deaths as the reference category (Table 2). Compared with individuals aged 65+, young adults (25–34 years) were less likely to die at home (OR, 0.41 [95% CI: 0.38–0.44]) or in hospice or nursing facilities (OR, 0.17 [95% CI: 0.15–0.19]). Notably, males had higher odds of dying at home (OR, 1.12 [95% CI: 1.10–1.15]) and in medical outpatient or ER settings (OR, 1.37 [95% CI: 1.30–1.45]) but lower odds of dying in hospice or nursing facilities (OR, 0.93 [95% CI: 0.91–0.95]) compared with women (Fig. 3). Racial differences in place of death were partially evident. Black individuals had similar odds of dying in outpatient or ER settings (OR, 0.99 [95% CI: 0.92–1.07]) compared with White individuals. However, they were significantly less likely to die at home (OR, 0.57 [95% CI: 0.55–0.59]) or in hospice or nursing facilities (OR, 0.50 [95% CI: 0.48–0.52]). Hispanic individuals had reduced odds of dying at home (OR, 0.73 [95% CI: 0.71–0.75]) or in hospice or nursing facilities (OR, 0.52 [95% CI: 0.50–0.53]) compared with non-Hispanics. Regional differences were also significant. Residents of large metropolitan areas (OR, 1.17 [95% CI: 1.09–1.25]) and medium-sized metropolitan areas (OR, 1.10 [95% CI: 1.02–1.18]) were more likely to die in medical outpatient or ER settings compared with rural counties (Fig. 4).

**Table 2:** Multivariable logistic regression between decedent characteristics and place of death (1999–2023).

Characteristics	OR (95% CI)	*P*-value
**Medical facility-outpatient or ER**		
Hispanics	0.993 (0.939 to 1.05)	0.803
Non-Hispanics	1.00	Reference
American Indians	0.143 (0.105 to 0.194)	<0.00001
Asians or Pacific Islanders	0.368 (0.274 to 0.494)	<0.00001
Blacks	0.996 (0.923 to 1.074)	0.913
White	1.00	Reference
Male	1.378 (1.308 to 1.451)	<0.00001
Female	1.00	Reference
25–34 years	1.297 (1.092 to 1.539)	0.003
35–49 years	2.332 (2.166 to 2.512)	<0.00001
50–64 years	1.862 (1.736 to 1.996)	<0.00001
65+ years	1.00	Reference
Large metro	1.173 (1.097 to 1.254)	<0.00001
Medium/small metro	1.101 (1.025 to 1.182)	0.008
Rural	1.00	Reference
Decedents home		
Hispanics	0.737 (0.719 to 0.755)	<0.00001
Non-Hispanics	1.00	Reference
American Indians	0.556 (0.524 to 0.589)	<0.00001
Asians or Pacific Islanders	0.477 (0.433 to 0.526)	<0.00001
Blacks	0.571 (0.551 to 0.591)	<0.00001
White	1.00	Reference
Male	1.128 (1.106 to 1.15)	<0.00001
Female	1.00	Reference
25–34 years	0.413 (0.386 to 0.441)	<0.00001
35–49 years	0.562 (0.548 to 0.576)	<0.00001
50–64 years	0.707 (0.693 to 0.722)	<0.00001
65+ years	1.00	Reference
Large metro	0.85 (0.83 to 0.872)	<0.00001
Medium/small metro	1.066 (1.04 to 1.094)	<0.00001
Rural	1.00	Reference
**Hospice or nursing facility**		
Hispanics	0.522 (0.506 to 0.539)	<0.00001
Non-Hispanics	1.00	Reference
American Indians	0.512 (0.475 to 0.552)	<0.00001
Asians or Pacific Islanders	0.131 (0.107 to 0.16)	<0.00001
Blacks	0.504 (0.484 to 0.526)	<0.00001
White	1.00	Reference
Male	0.932 (0.912 to 0.953)	<0.00001
Female	1.00	Reference
25–34 years	0.171 (0.154 to 0.19)	<0.00001
35–49 years	0.347 (0.337 to 0.358)	<0.00001
50–64 years	0.579 (0.566 to 0.592)	<0.00001
65+ years	1.00	Reference
Large metro	1.013 (0.984 to 1.043)	0.381
Medium/small metro	1.358 (1.318 to 1.4)	<0.00001
Rural	1.00	Reference

ER, emergency room; metro, metropolitan area; OR, odds ratio.

## Discussion

To our knowledge, this is the first study to comprehensively analyze national trends in ALC mortality and place of death in the U.S. from 1999 to 2023 using CDC WONDER. We observed a marked increase in ALC-related mortality over the study period, with the steepest rise during 2018–2021 followed by a subsequent decline. Place-of-death patterns also changed over time. Inpatient deaths decreased, whereas deaths at home and in the combined hospice/long-term care facility category increased. Sociodemographic differences were evident, with younger adults, men, racial and ethnic minority groups, and metropolitan residents showing different place-of-death patterns compared with their respective reference groups. These findings suggest persistent differences in end-of-life care pathways, although the observational design cannot establish causal mechanisms.

The rise in ALD-related mortality is consistent with national and international evidence showing an increasing burden of ALD. Julien *et al* projected a 77% increase in decompensated alcohol-associated cirrhosis by 2040, while CDC surveillance reported a 23% increase in ALD mortality between 2019 and 2020, coinciding with the first year of the COVID-19 pandemic^[^15,16^]^. Similarly, Kim *et al* observed sustained rises in mortality through 2023, particularly during the pandemic period^[^17^]^. Prior evidence describing disproportionately high ALD mortality among American Indian and Alaska Native populations, socioeconomic mortality gradients, and heavy alcohol use among patients with cirrhosis provides important context for the demographic differences observed in our study^[^18–20^]^. Pandemic-related increases in short-term ALD mortality, retail alcohol sales, transfer burden at liver centers, and policy changes facilitating alcohol home delivery and to-go sales may have further contributed to these trends^[^21–24^]^.

The decline in inpatient place of death should be interpreted in the context of broader changes in cirrhosis care, palliative care delivery, and pandemic-era care patterns. Laswi *et al* reported declining inpatient mortality among hospitalized patients with ALC from 1998 to 2018, which may reflect advances in the management of variceal bleeding, ascites, hepatorenal syndrome, spontaneous bacterial peritonitis, hepatic encephalopathy, and the timely use of antibiotics in cirrhosis with upper gastrointestinal bleeding^[^25–31^]^. At the same time, changes in palliative care consultations, telehealth-supported home care, and national shifts toward home and hospice deaths among Medicare beneficiaries provide additional context for the movement away from inpatient settings as places of death ^[^32–36^]^. Nonetheless, recent research indicates that while inpatient mortality has improved, long-term outcomes, specifically 1-year mortality, have not seen a comparable decline, suggesting a possible shift in mortality burden from the inpatient setting^[^37^]^.

Consistent with this pattern, our study observed an overall rise in home deaths and a mid-2010s peak in hospice and nursing facility deaths, with a decline thereafter, again indicating nonlinear change over time. A study conducted by Altaii *et al* in 2018 reported that between 2003 and 2015, home deaths rose from 21.6 to 26.1%, while hospice deaths increased markedly from 0.2 to 11.6% (*P* < 0.001)^[^6^]^. The recent multinational study by Lopes *et al* in 2024 confirmed that the pandemic years 2020–2021 were accompanied by a global increase in home deaths, including in the U.S., even after adjusting for expected pre-pandemic trajectories^[^38^]^.

These non-linear trends should also be interpreted within the broader U.S. health policy context. The Affordable Care Act (ACA) expanded Medicaid eligibility for many low-income adults, with the expansion commonly described as having an effective threshold of 138% of the federal poverty level, but implementation occurred at the state level and remained uneven across the country^[^39,40^]^. These coverage changes may have improved insurance coverage and access to serious-illness services for some low-income adults, and prior work has described the ACA as an important framework for improving end-of-life care access among patients with serious illness^[^41^]^. More directly, Han *et al* found that the ACA Medicaid expansion was associated with increased palliative care use among patients with advanced-stage cancers, supporting a plausible link between expanded coverage and greater access to supportive care services^[^42^]^. In this context, the mid-2010s rise in the proportion of ALC-related deaths occurring in hospice or nursing facility settings may be consistent with improved access to non-hospital end-of-life care pathways after ACA implementation, rather than an increase in mortality attributable to these settings. However, because CDC WONDER does not capture individual insurance status, Medicaid expansion exposure, hospice enrollment, or state-level policy implementation, these policy effects cannot be directly tested in the present analysis.

Despite these policy-level changes, several barriers continue to limit timely palliative and hospice care in cirrhosis. Prognostic tools such as the Model for End-Stage Liver Disease (MELD) and Child-Pugh scores, although central to assessing disease severity, are not routinely integrated into hospice eligibility, contributing to delayed referral and underutilization^[^43^]^. Beyond palliative and hospice care, access to advanced liver therapies such as transjugular intrahepatic portosystemic shunt (TIPS) and liver transplantation remains highly restricted and imposes a major financial burden. In a nationwide analysis of 4.9 million insured patients, Lange *et al* found that only 1% of patients with decompensated cirrhosis underwent liver transplantation and 5% received TIPS, despite a >50 % 1-year mortality and substantially higher healthcare costs compared with compensated patients^[^44^]^. Recent policy and practice developments, however, represent meaningful progress. Orman *et al* highlighted the need for standardized hospice referral pathways in cirrhosis^[^45^]^, and the AASLD 2022 Practice Guidance now recommends systematic incorporation of MELD-Na and structured advance care planning into routine management^[^46^]^. Most recently, in 2023, the Hospice in End-Stage Liver Disease (HELP) scale was validated in a large U.S. cohort by Brown *et al*, combining MELD-Na with key clinical variables (e.g., ascites, encephalopathy, frailty) to more accurately predict 6-month mortality and directly facilitate individualized hospice referral discussions^[^47^]^. These tools represent meaningful progress, yet implementation remains highly variable across care settings, highlighting persistent system-level gaps that likely contribute to the place-of-death disparities observed in our study.

Racial and socioeconomic differences in place of death observed in our study are consistent with the broader literature documenting unequal access to hospice, insurance coverage, and palliative services. Black individuals had lower odds of dying in the combined hospice/long-term care facility category, consistent with evidence that socioeconomic disadvantage, limited insurance coverage, reduced provider communication, and mistrust of medical institutions are associated with lower advance care planning among Black adults^[^48^]^. Insurance coverage may be an important contextual factor, as Hill *et al* demonstrated that Black adults under 65 remain 1.5 times more likely to be uninsured than White adults, particularly in non-Medicaid-expansion states^[^49^]^, and Nwankwo *et al* found even higher uninsured rates among Black immigrants^[^46^]^. These factors may contribute to delayed or reduced access to palliative and hospice services and provide context for the place-of-death differences observed in our study. The COVID-19 pandemic may have further influenced these patterns by straining institutional capacity and accelerating transitions to home discharge for non-COVID conditions, including cirrhosis^[^50–52^]^. Thus, the higher proportion of home deaths among Black individuals may be consistent with structural and socioeconomic barriers, fragmented access to supportive care, and differences in care pathways rather than cultural preference alone. These findings support further evaluation of policy and care-delivery strategies aimed at improving insurance coverage, culturally responsive care planning, and equitable end-of-life care.

Deaths in home, hospice, or nursing facility settings were least common among adults aged 25–34 years and most prevalent among individuals aged 65 years and older. The age-stratified pattern revealed an inverse association between age and the likelihood of dying in inpatient medical facilities. Younger adults with ALC were disproportionately more likely to die in hospitals, while older adults more frequently died at home or in hospice/nursing care settings. Our regression analysis reinforced this disparity: compared with decedents aged 65 years or older, those aged 25–34 years had significantly lower odds of dying at home or in hospice/ nursing homes. This pattern may be attributed to multiple factors. Younger adults with ALC often experience abrupt decompensation without prior engagement in palliative care or advance care planning^[^53,54^]^. Their deaths are more likely to occur in acute settings due to delayed recognition of terminal status, fewer social supports, and lower hospice enrollment rates^[^55^]^. In contrast, older adults are more likely to have established care plans, reside in long-term care settings, and qualify for Medicare-supported hospice services, facilitating death in non-acute environments^[^56^]^.

The disproportionate burden of ALC mortality among men, who comprised 72% of ALC deaths, corroborates existing literature documenting higher rates of heavy and binge drinking among men, as well as delayed health-seeking behaviors and reduced utilization of preventive care services^[^57–59^]^. Our multinomial regression further revealed that male decedents were more likely to die at home and in emergency or outpatient settings compared with women. This aligns with prior findings indicating men are less likely to engage with hospice or long-term care services, potentially due to social norms surrounding masculinity, caregiving, and perceived autonomy^[^60–62^]^. Moreover, men’s lower odds of dying in hospice or nursing facilities (OR, 0.93) may reflect underutilization of palliative services or delayed referrals to hospice care. Saito and colleagues identified a significant association between male sex and both delayed hospice admission and shorter hospice factors linked to poorer survival outcomes^[^63^]^. In contrast, women are generally more inclined to opt for end-of-life care and tend to align more closely with the objectives of palliative care^[^64^]^.

Our findings also indicate that patients with ALC living in large and medium metropolitan areas were more likely to die in outpatient or emergency settings than those in rural areas, suggesting geographic differences in place-of-death patterns. This pattern may be consistent with differences in hospice and palliative care availability, care transitions, and emergency care use across geographic settings^[^65,66^]^. Expanding hospice and home-based care in underserved rural communities, improving patient and family education, and facilitating smoother transitions from hospital to home or hospice care are critical steps to reduce preventable emergency deaths and promote equitable care delivery. Integrating telehealth and mobile palliative care teams has shown promise in overcoming geographic barriers and improving access to end-of-life care in rural populations^[^67^]^. Further research should focus on system-level barriers to palliative care access to inform policies tailored to the disparities.

## Limitations

Our study has certain limitations. It relies on death-certificate data abstracted from the CDC WONDER, which may not fully capture the complexity of end-of-life experiences among patients with ALC. The study’s focus on deaths with ALC listed as the underlying cause may exclude individuals with ALC who died from other causes, potentially leading to an underestimation of the actual burden of ALC-related end-of-life care. Additionally, aggregated de-identified data limit the ability to account for individual-level clinical factors, such as disease severity, comorbidities, or palliative care engagement, which are key determinants of care decisions and outcomes. This may obscure significant contextual differences in the circumstances surrounding deaths in various settings.

Furthermore, the database does not capture care transitions such as prior hospitalization or hospice enrollment, which may affect the interpretation of the place of death. Additionally, the database does not include variables for palliative care consultation or hospice enrollment (including home hospice). Therefore, we could not determine whether patients received palliative care consultation, were formally enrolled in hospice, or received home hospice services before death. In addition, the combined hospice/long-term care facility category cannot distinguish deaths in hospice facilities from deaths in nursing homes or long-term care facilities with or without hospice involvement. For this reason, this category should be interpreted as a place-of-death classification rather than as a direct measure of hospice utilization or palliative care access. The absence of behavioral or psychosocial data, including alcohol use patterns, mental health, or social support systems, also limits the ability to explore critical upstream factors that influence end-of-life outcomes. Moreover, the mortality data do not provide insight into the root causes of sociodemographic disparities in place of death, such as differences in health care access, quality of care, or cultural preferences, which limits the ability to conclude causation across race, sex, urbanization level, or geographic region. While individual socioeconomic and educational variables were not captured in the dataset, their influence is likely reflected indirectly through broader demographic and geographic patterns. These unmeasured social determinants are important contextual factors that can further shape access to palliative and hospice services; recognizing them complements rather than limits the robustness of our findings.

## Conclusion

This national analysis of more than two decades of data shows substantial shifts in where adults with ALC die in the U.S. Inpatient deaths declined over time, whereas deaths at home and in the combined hospice/long-term care facility category increased, suggesting evolving place-of-death patterns. Significant associations by age, sex, race, ethnicity, and urbanization indicate that end-of-life care pathways differ across sociodemographic groups. These findings are consistent with uneven access to supportive care and advance care planning, and they support continued efforts to improve palliative care access, culturally responsive care planning, and transitions across care settings.

## Data Availability

The datasets generated and analyzed during the present study are publicly available on the Centers for Disease Control and Prevention’s Wide-ranging Online Data for Epidemiologic Research (CDC WONDER) platform at https://wonder.cdc.gov/. All data are open-access, de-identified, and freely available for public use.
